# Over-expression of BAG-1 in head and neck squamous cell carcinomas (HNSCC) is associated with cisplatin-resistance

**DOI:** 10.1186/s12967-017-1289-2

**Published:** 2017-09-06

**Authors:** Shutong Liu, Bo Ren, Hang Gao, Suchan Liao, Ying-Xian Zhai, Shirong Li, Xue-Jin Su, Ping Jin, David Stroncek, Zhixiang Xu, Qinghua Zeng, Yulin Li

**Affiliations:** 1 The Key Laboratory of Pathobiology, Ministry of Education, Norman Bethune College of Medicine, Jilin University, Changchun, 130021 Jilin China; 2 Cell Processing Section, Department of Transfusion, Clinical Center, National Institutes of Health, Bethesda, MD 20892 USA; 3 Department of Physiology, Youjiang Medical University for Nationalities, Baise, 533000 Guangxi China; 4 Division of Hematology/Oncology, Department of Medicine, University of Alabama at Birmingham, Birmingham, AL 35294 USA

**Keywords:** Head and neck squamous cell carcinomas, Cisplatin, Drug resistance, Biomarker, BAG-1, BCL-xL

## Abstract

**Background:**

In order to improve therapy for head and neck squamous cell carcinoma (HNSCC), biomarkers associated with local and/or distant tumor relapses and cancer drug resistance are urgently needed. This study identified a potential biomarker, Bcl-2 associated athanogene-1 (BAG-1), that is implicated in HNSCC insensitive to cisplatin and tumor progression.

**Methods:**

Primary and advanced (relapsed from parental) University of Michigan squamous cell carcinoma cell lines were tested for sensitivity to cisplatin and gene expression profiles were compared between primary (cisplatin sensitive) and the relapsed (cisplatin resistant) cell lines by using Agilent microarrays. Additionally, differentially expressed genes phosphorylated AKT, and BAG-1, and BCL-xL were evaluated for expression using HNSCC tissue arrays.

**Results:**

Advanced HNSCC cells revealed resistant to cisplatin accompanied by increased expression of BAG-1 protein. siRNA knockdown of BAG-1 expression resulted in significant improvement of HNSCC sensitivity to cisplatin. BAG-1 expression enhanced stability of BCL-xL and conferred cisplatin resistant to the HNSCC cells. In addition, high levels of expression of phosphorylated AKT, BAG-1, and BCL-xL were observed in advanced HNSCC compared to in that of primary HNSCC.

**Conclusion:**

Increased expression of BAG-1 was associated with cisplatin resistance and tumor progression in HNSCC patients and warrants further validation in larger independent studies. Over expression of BAG-1 may be a biomarker for cisplatin resistance in patients with primary or recurrent HNSCCs and targeting BAG-1 could be helpful in overcoming cisplatin resistance.

**Electronic supplementary material:**

The online version of this article (doi:10.1186/s12967-017-1289-2) contains supplementary material, which is available to authorized users.

## Background

Head and neck squamous cell carcinomas (HNSCC) are the fifth most common non-skin cancer worldwide and the third most common cancer in developing countries [1, 2]. HNSCC constitutes up to 90% of all head and neck cancers with an annual incident of 600,000 cases and its overall 5 year survival rate is only 40–50% despite aggressive treatment [3]. Cisplatin is one of the most common chemotherapeutics being used as a first-line agent in the treatment of HNSCC. Cisplatin exerts its anti-tumor effects through the generation of unrepairable DNA lesions that result in cellular apoptosis via the activation of DNA damage response [4, 5]. Resistance to cisplatin is a major obstacle to effective cancer therapy because clinically relevant levels of resistance emerge quickly after treatment. Many important signaling pathways, which regulate the expression of genes controlling growth, survival, and chemosensitivity, are involved in development of cisplatin resistance, including mutation or loss of function of tumor suppressor genes such as p53 as well as the over expression, and activation of oncogenic proteins such as HER2, Aurora-A, and members of the BCL-2 family [3–11]. It is essential to improve the efficacy of cisplatin therapy using a mechanism-based approach, so it is urgent to identify the critical molecules and signaling pathways that underlie the development of cisplatin resistance.

B-cell lymphoma 2-associated athanogene-1 (BAG-1), is a multifunctional protein that regulates a variety of cellular processes: proliferation, cell survival, transcription, apoptosis, and motility [12]. BAG-1 has three isoforms which are produced by the alternative translation initiation of a single mRNA transcript that results in different N-terminus regions. BAG-1 isoforms appear to be differentially localized in cells. BAG-1L is a 50 kDa protein that is localized to the nucleus due to the presence of a nuclear localization signal (NLS). In contrast, a shorter isoform of BAG-1, BAG-1s (36 kDa), exists in the cytoplasm and an intermediate sized isoform, BAG-1M (46 kDa), partitions between the cytoplasm and nucleus via interactions with companion proteins [13]. Interactions of BAG-1 with various proteins(s)/complexes determines its function in the cell. Well-known interacting partners of BAG-1 isoforms are, BCL-2, Raf-1, Hsc70/Hsp70 system, nuclear hormone receptors (NHR), ubiquitin/proteasome machinery and DNA [14].

The B-cell lymphoma 2 (BCL-2) protein family is a group of structurally related proteins have opposite functions, and can be classified into two functional subgroups [15, 16]: Anti-apoptotic proteins including BCL-2, BCL-xL, BCL-W, MCL-1, BCL-B, protect cells from cytotoxic insults such as chemotherapeutic medicine [17]; Pro-apoptotic proteins, such as BID, BIM, BAD, BAC, BAK. Although BCL-2 protein was investigated in various of cancers apoptosis studies [18], BCL-xL, a protein encoded by gene BCL2L1, is considered as a more effective marker than BCL-2 [19].

Currently there are no defined targetable genetic aberrations for HNSCC, and no approved therapies are tied to genetic alterations [20, 21]. All patients with HNSCC are treated with a largely uniform approach based on stage and anatomic location, typically using surgery, radiation therapy, and chemotherapy alone or in combination [20, 21]. Cetuximab, an anti-EGFR antibody, is the only approved targeted therapy for HNSCC with a single-agent response rate of 10–13%. Despite the modest response rate, there are no validated predictive biomarkers for benefit from cetuximab therapy [22, 23].

Previous gene expression studies of other cancers have produced lists of differently expressed genes but have failed to establish how these genes form regulatory networks [22–24]. Systematic examination of datasets for genes and pathways associated with cisplatin-resistant has been limited. Moreover, these studies have ignored genes that do not pass randomly or empirically determined criteria for gene selection. Therefore, we adopted a computational tool, Ingenuity Pathway Analysis (IPA; Ingenuity Systems, Mountain View, CA), to visualize regulatory networks of differentially expressed genes and the corresponding canonical pathways that govern the response to cisplatin treatment.

In this study, we combined microarray technology and IPA, to identify and validate genes with altered expression in cisplatin resistant University of Michigan Squamous Cell Carcinoma (UMSCC) laryngeal cells. We have found that BAG-1 is a gain function gene associated with cisplatin resistance. We also discuss possible individualization of cancer chemotherapy with possible new molecular markers of anticancer resistance.

## Methods

### Study design

First, we screened UMSCC cell lines for resistance to cisplatin by viability, proliferation by MTT assay. Then we compared resistant and non-resistant cells by gene expression analysis. Next, we confirmed expression of differentially expressed genes in UMSCC cell lines at the protein level by western blot and immunohistochemistry. To further prove our hypothesis, we used the UMSCC cells with three specific inhibitors and siRNA.

### Cells and cell culture

UMSCC cells were kindly provided by Dr. Thomas Carey (Department of Otolaryngology/Head and Neck Surgery, University of Michigan, MI). UMSCC cells were maintained in Dulbecco’s modified Eagle’s medium (DMEM) supplemented with 10% fetal bovine serum (FBS), l-glutamine, sodium pyruvate, nonessential amino acids (Life Technologies, Inc.). Adherent monolayer cultures were maintained on plastic plates and incubated at 37 °C in 5% CO_2_ condition. The cultures were mycoplasma-free and maintained for no longer than 12 weeks after they were recovered from frozen stocks.

### Preparation of reagents

Cisplatin was purchased from Sigma-Aldrich and was diluted in phosphate buffered saline (PBS) immediately before each experiment. Propidium iodide and 3-(4,5-dimethylthiazol-2-yl)-2,5-diphenyl-tetrazolium bromide (MTT) were purchased from Sigma-Aldrich. Stock solutions were prepared by dissolving either 0.5 mg of propidium iodide or 2 mg of MTT in 1 mL of PBS. Each solution was filtered, protected from light, stored at 4 °C, and used within 1 month. Bag-1 siRNAs (h) were purchased from Santa Cruz Biotechnology (sc-29211). Specific inhibitors ly29004 (#9901) and U0126 (#9903) were purchased from Cell Signaling, NSC 74859 was purchased from R&D (cat#4655).

### Cell proliferation assay

The anti-proliferative activity of cisplatin against UMSCC HNSCC cells in vitro was determined by MTT cell viability assay. Briefly, UMSCC 14A, B and UMSCC 17A, B cells were plated in 96-well plates in medium. After a 24-h attachment period, the cells were incubated for indicated hours in various concentrations of cisplatin or with PBS alone as a control. Cells were then incubated in medium containing 10% FBS and 0.25 mg/mL MTT for 3 h. The cells were then lysed in 100 μL dimethylsulfoxide to release formazan. We used an EL-808 96-well plate reader (BioTek Instruments) set at an absorbance of 570 nm to quantify the conversion of MTT to formazan. The experiment was repeated in triplicate.

### Clonogenic survival assay

To determine the sensitivity of the HNSCC cells to cisplatin, we performed a clonogenic survival assay. Cells in culture were treated with the indicated concentrations of cisplatin for 2 h or with PBS alone as a control after which they were cultured for 12 days in medium without cisplatin. The cell medium was changed with fresh new medium every 3 days. At the end of experiment, the cells were stained with 0.5% crystal violet in absolute ethanol, and colonies with more than 50 cells were counted under a dissection microscope.

### Agilent microarrays

Total RNA was extracted from each cell line using miRNA Easy Kit (Qiagen, Germantown, MD) and RNA was evaluated using NanoDrop 2000 (Thermo Scientific, Wilmington, DE). Microarray expression experiments were performed on 4 × 44 K whole human genome microarray (Agilent technologies), according to the manufacturer’s instructions, the images of arrays were scanned by using Agilent Scan G2505B and then following the data extraction by using the Feature Extraction Software (Agilent Technologies, GE2-1200_Jun14). Partek Genomic Suite 6.6 (Partek Inc., St. Louis, MO, USA) was used for data visualization, identification of differentially expressed transcripts and hierarchical cluster analysis. Fluorescence intensity data was transformed to log 2 ratios of each sample versus the universal human RNA reference (Stratagene, Santa Clara, CA, USA). Then t-tests were used to identify differentially expressed genes. Analysis of functional pathways was performed by ingenuity pathway analysis (IPA) tool (Ingenuity System Inc., Redwood City, CA, USA). The microarray data had been deposited in National Center for Biotechnology Information Gene Expression Omnibus database GSE102787).

### Western blotting

The cultured cells were analyzed by western blotting. UMSCC cells (2 × 10^6^ per well) were plated in 100 mm dishes (Costar) in 10 mL medium containing 10% FBS, incubated for 24 h, and then treated with indicated concentration of cisplatin, and PBS as untreated control. Total cell lysates were then obtained and subjected to Western blot analysis as previously described [25]. The membranes were blocked for 1 h at room temperature with 5% bovine serum albumin in 0.1% Tween 20 in Tris-buffered saline and incubated overnight at 4 °C with anti-BAG-1 (sc-939 1:500), anti-BCL-xL (sc-7195 1:1000), anti-BCL-2 (sc-7382, 1:1000), anti-Akt (Cell Signaling; 1:1000), anti-phosphorylated Akt (Ser473; Cell Signaling; 1:1000), anti-mitogen–activated protein kinase (MAPK, Cell signaling, 1:1000) in 5% non-fat milk in 0.1% Tween 20 tris-buffered saline. Next, the membranes were washed with 0.1% Tween 20 in tris-buffered saline and incubated for 1 h at room temperature in horseradish peroxidase-conjugated anti-rabbit immunoglobulin G (Santa Cruz Biotechnology) to detect EGFR, phosphorylated EGFR, or species-appropriate fluorescently conjugated proteins (goat anti-rabbit IRDye 800 and goat anti-mouse IRDye 800, Invitrogen). The membranes were then analyzed using the SuperSignal West chemiluminescent system (Pierce Biotechnology). To verify equal protein loading, we stripped and re-probed the membranes with anti-GAPDH (sc-47724, 1:5000).

### HNSCC tissue array immunohistochemistry for BAG-1 and BCL-xL expression

Immunohistochemical studies of BAG-1, BCL-xL, and phosphorylated AKT (Ser473) were performed on both paraffin-embedded tissue sections from HNSCC tissues arrays (IMH-310), which were purchased from IMGENEX (San Diego, CA). HNSCC tissue arrays contained 58 cases of primary HNSCC and 2 cases of metastatic HNSCC mounted on slides. The tissue arrays were deparaffinized by xylene, and then, re-hydrated with sequential washes of 100%, 75%, 50% ethanol, and PBS. For antigen retrieval, slides were placed in 50 mM Tris–HCl buffer pH 9.0, heated in a decock pressure cooker for 20 min, and then stayed in the buffer for 15 min. Endogenous peroxidase activity was inhibited with 3% hydrogen peroxidase in PBS. Non-specific binding was blocked with 3% normal goat serum for 30 min. Tissue sections arrays were then incubated with anti-BAG-1 or anti-BCL-xL antibodies (Santa Cruz Biotech; Santa Cruz, CA), phosphorylated AKT (Cell signaling) for 1 h at room temperature. Immunodetection was performed using DAB staining systems according to manufacturer’s instructions (ScyTek Laboratories; Logan, UT 84321). All sections were counterstained with haematoxylin. After dehydration with washes of 95 and 100% ethanol and xylene, tissue sections and tissue arrays with permanent mounting medium were covered with glass coverslips, and viewed by light microscope.

## Results

### UMSCC cells response to cisplatin

**Fig. 1:**
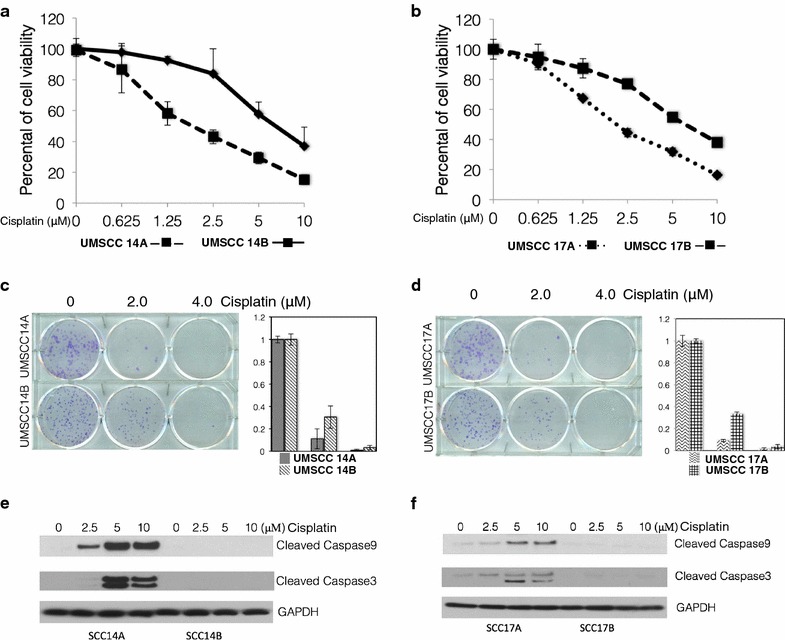
UMSCC cells respond to Cisplatin challenge. **a**, **b** Cell viability MTT assays were performed in UMSCC 14A, 14B and 17A, 17B cells after exposure to a serial dose–response of cisplatin for 24 h. **c**, **d** (Two-tailed Student’s *t*-test, p < 0.05). **c**, **d** Clonogenic cell survival assays in UMSCC 14A, 14B and 17A, 17B cells after the cells were treated with cisplatin for 2 h with 2.0 and 4.0 μM of cisplatin (two-tailed Student’s *t*-test, p < 0.05). **e**, **f** Whole-cell lysate samples from UMSCC 14A, 14B and 17A, 17B cells treated with indicated concentration of cisplatin were used for western blot and probed with monoclonal antibodies for cleaved caspase 9 and cleaved caspase 3, each membrane was stripped and re-probed with GAPDH

In order to study cisplatin resistance in head and neck cancer, we screened a panel of UMSCC cell lines (cell line information as shown in Additional file 1: Table S1). We measured the cisplatin sensitivity of pairs of primary UMSCC cells (A’s) and their advanced UMSCC cells (B’s). We found that advanced UMSCC cells, 14B and 17B, were more resistance to cisplatin, as measured by cell viability, than their primary cells, 14A and 17A, respectively (Fig. 1a, b). The primary UMSCC cells were relatively sensitive to cisplatin treatment with an IC_50_ of 1.56 μM and 1.85 μM, whereas the advanced UMSCC cells 14B and 17B were more resistant to cisplatin treatment, with an approximately threefold decrease in sensitivity compared to the primary UMSCC cells 14A and 17A; 4.85 μM for 14B and 5.5 μM for 17B.

In addition, the UMSCC cells were tested in a cell proliferation study. We evaluated the effects of cisplatin on the same cells and found that clonogenic survival after treatment with cisplatin was markedly different between primary UMSCC cells 14A and 17A and advanced UMSCC cells 14B and 17B. Figure 1c and d demonstrate significantly more clonogenic proliferation in both advanced UMSCC 14B and 17B cell lines compared with their primary cell counterparts.

To confirm the cell proliferation data, we evaluated the effects of cisplatin on induction of cell apoptosis by measuring activated caspases using a western blot assay. As shown in Fig. 1e, f, after 24 h of cisplatin treatment, both primary UMSCC 14A and 17A cells showed a dose dependent induction of cleaved caspase 9 and caspase 3, but the advanced UMSCC 14B, and 17B cells showed no detectable cleaved caspase 9, and caspase 3. These data indicated that advanced UMSCC 14B and 17B cells are more resistant to cisplatin compared to their primary counterpart cells.

### Gene array data

Initially, we studied molecular signatures of local and/or distant tumor relapses of HNSCC. RNA and DNA from seven pairs of UMSCC cell lines were purified and subjected to gene array analysis using Agilent Whole Human Genome microarray (Additional file 2: Figure S1A). Then t-tests were used to identify differentially expressed genes between relapsed and primary cell lines, and a set of 739 genes (unadjusted p value <0.05, Additional file 3: Table S2) was selected for genetic network and functional analysis which was performed by Ingenuity Pathway Analysis as shown in Additional file 2: Figure S2B.

**Fig. 2:**
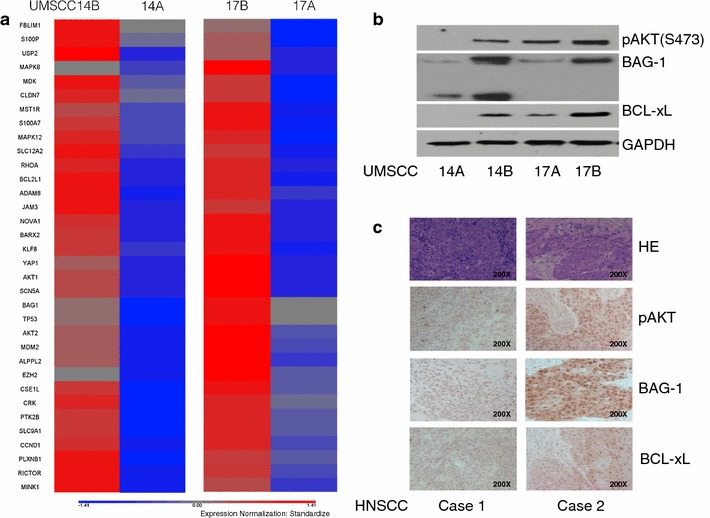
Validation of up-regulated genes from genes array analysis of genes differently expressed in cisplatin resistant UMSCC cells via western blot and tissue array. **a** RNAs from UMSCC 14A, 14B and 17A, 17B cells were purified and requested for the gene array analysis. Up-regulated genes in both UMSCC 14B and 17B cells (associated with drug resistant) were listed. **b** Whole-cell lysate samples from UMSCC 14A, 14B and 17A, 17B cells were used for western blot and probed with antibodies for phosphorylated AKT, BAG-1, and BCL-xL. Each membrane was stripped and re-probed with GAPDH for loading control. **c** Immunohistochemistry staining of phosphorylated AKT, BAG-1, and BCL-xL on HNSCC tissue array. The imagines show representative staining patterns of AKT, BAG-1, and BCL-xL, in the primary HNSCC (Case 1) and metastatic HNSCC (Case 2), the imagines of HE staining were downloaded from online data of IMGENEX

Next, we focused on identifying the main gene expression changes associated with cisplatin resistance in the advanced UMSCC cells (14B and 17B) cells compared with their primary (14A and 17A) cells. We performed global gene expression analysis of these two pairs UMSCC cell lines using Agilent Whole Human Genome microarray. We then compared the average fold changes of expressed genes for the two pairs of cell lines and identified genes differentially expressed by 2.0 or more-fold and were either up- or down-regulated. As listed in Fig. 2a, the genes are associated with tumor progression and invasion based on known functions in the IPA Diseases and Functions annotation. The differentially expressed genes also included AKT1, AKT2 and TP53 which are associated with drug resistance. BAG-1 which has been reported to form a complex with B-Raf and AKT at the mitochondrial membrane to regulated bad phosphorylation, IAP (inhibitor of apoptosis protein) expression and cell survival [26, 27]. It has also been reported that BAG-1 in association with HGF receptor prevents cell death [28]. We have previous reported that when HGF activates its receptor it initiates down-stream signaling cascades including PI3k/AKT and MAPK/ERK pathways which render HNSCC cell resistance to cell death and induce progression [25, 29, 30]. This suggests that it would be of interest to study the coordinated expression of BAG-1 and AKT in HNSCC cells. We found that the expression of both BAG-1 and AKT correlated with cisplatin resistance in both pairs of UMSCC cells 14A, B, and 17A, B).

### Confirmation of expression of the three genes in cisplatin-resistant UMSCC cells

Next, we investigated the expression of AKT and BAG-1 in UMSCC cells at the protein level. Since BAG-1 in known to interact with BCL-2 proteins, we also assessed the expression of BCL-xL. We hypothesized that the up-regulation of these genes or proteins in advanced UMSCC cells were required for cisplatin resistance. Increased expression of phosphorylated AKT, BCL-xL, Bag-1 were observed by western blotting in cisplatin-resistant UMSCC 14B, and 17B cells (Fig. 2b). These results confirmed the up regulation of the three genes noted in the gene expression arrays in the advanced cisplatin-resistant UMSCC 14B and 17B cells compared to the primary UMSCC 14A and 17B cells.

### Immunohistochemistry (IHC) study of HNSCC tissue arrays

To examine the protein expression pattern of the three genes in clinical HNSCCs, we selected IMGENEX (San Diego, CA) HNSCC tissues arrays (60 cases/slide, IMH-310) for IHC staining of the three proteins. Although some of the tissues showed no signal (maybe due to the quality of the samples), Fig. 2c shows representative results of phosphorylated AKT, BCL-xL, Bag-1 expression in the primary HNSCC (Case 1) and metastatic HNSCC (Case 2). The results implied that these proteins are associated with HNSCC progression and are potential cisplatin resistant proteins. Only two cases of metastatic HNSCCs were among the 60 cases included in these tissue arrays and more clinical cases needed to be studied for further confirmation.

### Contribution of BAG-1 to the UMSCC cells cisplatin resistance

**Fig. 3:**
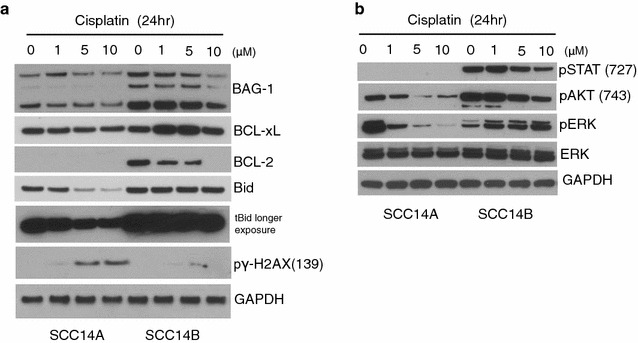
Sustained high expression of BAG-1, and its associated proteins in response to cisplatin resistance. Whole cell lysates of UMSCC 14A, 14B and 17A, 17B cells were treated with indicated concentration of cisplatin or absence for 24, for western blot analysis. **a** Membranes were probed with antibodies for BAG-1, BCL-xL, BCL-2, BID, and phosphor γH2AX. **b** Whole cell lysates were also probed for pro-survival pathway, PI3K/AKT, Jak/STAT3, and MAKP/ERK. Each membrane was stripped and re-probed with GAPDH for loading control

Since BAG-1 protein is frequently expressed in various of cancers [31–34], including HNSCC [35, 36], it is intriguing to see whether a longer duration of high BAG-1 expression is associated with cisplatin resistance of UMSCC cells. As the results show in Fig. 1, cisplatin induced cleavage of caspase 9 and caspase 3 in both of UMSCC 14A, and 17A cells, but not in the advanced UMSCC 14B, and 17B cells. These data prompted us to evaluate the expression of BAG-1 as well as other cell survival related proteins in the UMSCC 14B, and 17B cells. As shown in Fig. 3a, after 24 h BAG-1 expression was maintained at a high level in the cisplatin resistant UMSCC 14B cells, and BAG-1 expressing level remained lower level in UMSCC 14A cells. Interestingly, BCL-xL protein expression showed the same pattern as BAG-1 in the both cells. Even after 24 h we could not detect BCL-2 protein in the UMSCC 14A cells but higher level of BCL-2 expression was found in UMSCC 14B cells. However, no BCL-2 protein expression was detected in UMSCC 14B cells incubated with highest dose of ciplatin (10 μM) treatment. Bid protein, a member of the pro-cell death BCL-2 family, was cleaved (tBid) in UMSCC14A cells but it was not detected in the UMSCC 14B cells. It was well documented that cisplatin targets cell DNA to form a variety of cross links and monoadducts, which contribute to cytotoxicity by blocking DNA replication and inducing apoptosis [5, 6]. γH2AX is a subtype of histone H2A. In the process of DNA double-strand breaks (DSBs) repair, the γH2AX 139^Ser^ site is phosphorylated. Histone γH2AX phosphorylation is a sensitive marker for DNA DSBs and associated with resistance to multiple chemotherapy drugs including cisplatin [37]. In Fig. 3a, the expression level of phosphorylated γH2AX (s139) was significantly higher in UMSCC 14A cells than that in UMSCC 14B cells indicating that cisplatin caused more severe DNA damage in UMSCC 14A cells than that in UMSCC 14B cells.

### BAG-1 associated pro-survival proteins in UMSCC resistant cells

It is interesting to note that both BAG-1 and BCL-xL expression was maintained at a high level for the whole time course of cisplatin challenging in the cisplatin resistant UMSCC 14B cells. These results led us to exploit other proteins, which may play a role in involving cisplatin resistance. As shown in Fig. 3b, expression patterns of phosphorylated AKT, phosphorylated ERK, and phosphorylated STAT3 all remained at high levels in UMSCC 14B cells but not in the UMSCC 14A cells. PI3K/AKT, MAPK/ERK, Jak/STAT signaling pathways are involved in cell survival and proliferation. We have reported that up-regulated PI3K/AKT and MAPK/ERK signaling pathways were associated with HNSCC progression [25, 30]. STAT3 is an oncogene that is overexpressed in majority of HNSCCs. Activation of STAT3 leads to proliferation and survival mediated by the induction of specific target genes, such as cyclin D1, Bcl-2, and BCL-xL [38]. We and others have reported that interruption of STAT3 impedes cancer cell growth and enhances apoptosis in HNSCC [39, 40].

**Fig. 4:**
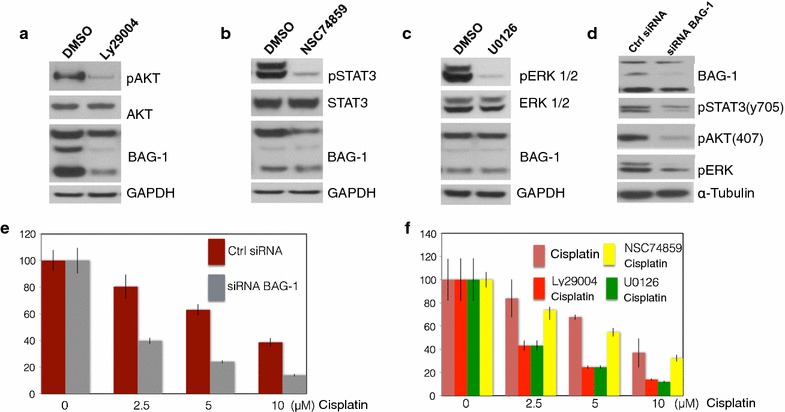
BAG-1 interacted with pro-survival signaling pathways in HNSCC cells. **a** Inhibition of PI3K/AKT by ly29004 resulted in decreased phosphorylated AKT and BAG-1 expression in the cells by not the normal AKT and loading control GAPDH. **b** Inhibition of Jak/STAT3 by NSC 74859 resulted in decreased phosphorylated STAT3A and BAG-1 expression in the cells by not the normal STAT3 and loading control GAPDH. **c** Inhibition of MAKP/ERK by U0126 resulted in decreased phosphorylated ERK but no effect on BAG-1, normal ERK, and loading control GAPDH. **d** siRNA BAG-1 knockdown BAG-1 expression in UMSCC 14B cells resulted in decreased expression phosphorylated AKT, phosphorylated STAT3, and phosphorylated ERK, but not the loading control α-Tubulin. **e** siRNA BAG-1 knockdown BAG-1 in the cells lead UMSCC 14B cells sensitive to cisplatin than that of shRNA control UMSCC 14B cells. **f** Specific inhibitors Ly29004 (p < 0.05) and NSC74859 (p < 0.05) enhanced cisplatin inhibition effect on UMSCC 14B cell proliferation, respectively. Not significant enhanced effect was found for the U0126 treated cells (p > 0.05)

We investigated the effects of AKT, ERK, and STAT activity on BAG-1 expression in UMSCC cells. As shown in Fig. 4a, b, specific inhibitors targeting PI3K/AKT (ly29004, Cell Signaling, #9901) and Jak/STAT3 (NSC 74859, R&D cat#4655) activation resulted in decreased expression of BAG-1 protein in UMSCC 14B cells compared to that in the control cells, but there were no significant changes in BAG-1 protein expression in the cells following inhibition of MAKP/ERK (U0126, Cell Signaling, #9903) (Fig. 4c). Intriguingly, siRNA knock down of BAG-1 in the UMSCC 14B cells resulted in a decreased expression of phosphorylated AKT, phosphorylated ERK, and phosphorylated STAT3 in the UMSCC 14B cells compared to control cells (Fig. 4d). These results indicate that BAG-1 may interact with various pro-survival proteins involved in cisplatin resistance indicating that it has multiple functions. To see whether knockdown of BAG-1 would render the cell vulnerable to cisplatin challenge, we compared the cell viability between the control siRNA and the siRNA BAG-1 treated UMSCC 14B cells by MTT assay. As expected UMSCC 14B cells with BAG-1 knockdown were much more susceptible to cisplatin compared to its control cells (Fig. 4e). Inhibition of PI3K/AKT by ly29004 and Jak/STAT3 by NSC 74859 resulted in a significant decrease of cell viability of UMSCC 14B cells compared to that of cisplatin treated UMSCC 14B cells, respectively (Fig. 4f), but no significant difference was seen in U0126 treated the UMSCC 14B cells (Fig. 4f). These results suggested that BAG-1 may associate with PI3K/AKT and Jak/STAT3 pathways involved in HNSCC cisplatin resistance.

## Discussion

Patients with HNSCC are treated aggressively with surgery followed by radiation and often with cisplatin [24]. Although these treatments increase loco-regional control, there are frequently disfiguring and induce high-grade toxicities limiting their effectiveness [41]. Furthermore, resistance to cisplatin and radiation contributes to tumor recurrence, and options for those who do not respond are limited to palliative care. Targeted therapies for HNSCC are currently limited to experimental agents targeting the EGF receptor [42]. We used the two pairs of cell lines (each pair of cells were established from the same UMSCC patient) for genome-wide expression analysis to identify cisplatin-resistance candidate genes. Moreover, because previous microarray studies have produced lists of differentially expressed genes [43] and ignored the genes that did not pass randomly or empirically determined criteria for gene selection, we adopted a computational tool, IPA, to visualize regulatory networks and gene ontology of differentially expressed genes. We identified BAG-1 genes as differentially expressed in the two cisplatin-resistant UMSCC cell lines. We found BCL-xL and AKT were also among the up-regulated genes accompany with BAG-1. This indicates that the ability of HNSCC cells to gain cisplatin resistance is multifactorial and that several mechanisms are encountered simultaneously within the same tumor cells. Therefore, we believe that the genes selected using our microarray approach to be new candidate cisplatin-resistant genes in HNSCC. Tumor response to cisplatin resistance cannot be predicted by one factor and may be determined by a critical balance of expression of several genes. Our selected genes might be helpful in the development of individualized HNSCC chemotherapy. This is likely to have an impact on current clinical practice for eligibility for chemotherapy in patients with HNSCC. The biological basis for the association between high BCL-xL and BAG-1 expression and cisplatin resistance in HNSCC has yet to be determined. Given that both of these proteins are key anti-apoptotic mediators that are part of the mitochondrial (indirect) pathway [44], it suggested that targeting BAG-1 and/or BCL-xL might be an effective adjuvant therapy in a subset of HNSCCs in future.

The two major forms of cisplatin resistance are intrinsic resistance, in which previously untreated tumor cells are inherently insensitive to the chemotherapeutic agent, and acquired resistance, in which treated tumor cells become insensitive after drug exposure. The various mechanisms of cisplatin resistance have been studied hoping to overcome this major chemotherapeutic obstacle. Research has determined that acquired cisplatin resistance is multifactorial, in that it involves host factors, genetic and epigenetic changes, and numerous molecular events [45]. Resistance itself may be due to decreased drug accumulation, alteration of intracellular drug distribution, reduced cell-cycle deregulation, increased damaged DNA repair and a reduced apoptotic response [46]. It was reported that over expression of the multi drug resistant gene (MDR1) is associated with drug-resistant cancer cells. However, little is known about the genes differentially expressed in cisplatin-resistant HNSCC cells [43]. Recently developed techniques for genome-wide expression analysis hopefully will provide additional information, novel candidate genes associated with cancer drug resistance, and perhaps new therapeutic targets.

The identification of pretreatment molecular markers that can predict response to therapy is of great interest in head and neck oncology and is required to develop personalized treatments that maximize survival while minimizing morbidity. Several studies have been performed on drug sensitivity and drug resistance in untreated human cancer cell lines and drug-exposed cells using a gene expression microarray technologies [47–49]. These studies showed correlations between gene expression and drug activity and the genes differentially expressed in drug-sensitive and drug-resistant cancer cells. In addition, several gene expression microarray studies have been performed to identify genes with altered expression in HNSCC [45, 50]. From these studies, numerous genes have been associated with the development and progression of head and neck cancer, some of which will be used as novel chemotherapeutic targets to treat or prevent HNSCC.

It had been theorized that each cancer cell represents a different pattern of drug-resistant gene expression signature, even within cells clonally derived from the same cancer, and may be expected to exhibit considerable heterogeneity with respect to drug resistance [45]. Here, we still suggest that targeting BAG-1 and/or BCL-xL in HNSCCs might improve the therapeutic ration of adjuvant therapy in a subset of HNSCCs.

## Conclusions

In summary, we found that BAG-1 in association with BCL-xL and AKT genes were differentially expressed in the two cisplatin-resistant UMSCC cell lines. Tissue array analysis and in vitro studies suggested that up-regulation of BAG-1 along with BCL-xL was associated with cisplatin resistance for HNSCC and targeting BAG-1 and/or BCL-xL in HNSCCs might overcome cisplatin resistance in a subset of patients.

## Additional files

**Additional file 1: Table S1.** Information of Seven pairs of UMSCC cell lines. These pairs of cell lines were established from the same individual HNSCC patient. A represents the cell line was established from primary or first time excision, B represents the cell line was established from advanced or secondary excision.

**Additional file 2: Figure S1.** Differential expression analysis of relapsed and local cell lines. T-tests were used to identify differentially expressed genes between relapse and primary cell lines. 739 genes (unadjusted p value <0.05) were shown.

**Additional file 3: Table S2.** Gene array analysis of altered gene expression in advanced UMSCC cells. A. RNA and DNA were purified from seven pairs of UMSCC cell lines for gene array analysis and validation of interested genes. B. List of top diseases and bio functions generated by Ingenuity Pathway Analysis software.

## Abbreviations

HNSCC: head and neck squamous cell carcinomas
UMSCC: University of Michigan squamous cell carcinoma
BAG-1: B cell lymphoma 2-associated athanogene-1
BCL-2: B-cell lymphoma 2
BCL2L1: B-cell lymphoma 2 like 1
BCL-xL: B-cell lymphoma-extra large
BID: BH3 interacting domain death agonist
tBid: truncated BH3 interacting domain death agonist
MDR1: multi drug resistant gene
IPA: ingenuity pathway analysis
DSBs: DNA double-strand breaks
MTT: 3-(4,5-dimethylthiazol-2-yl)-2,5-diphenyl-tetrazolium bromide
PI: propidium iodide
