# Supplementary material for: Over-expression of BAG-1 in head and neck squamous cell carcinomas (HNSCC) is associated with cisplatin-resistance
Source: J Transl Med. 2017 Sep 6;15:189. doi: 10.1186/s12967-017-1289-2 (PMC5588726; doi:10.1186/s12967-017-1289-2)
Supplement: Supplementary file 1 — Additional file 1: Table S1. Information of Seven pairs of UMSCC cell lines. These pairs of cell lines were established from the same individual HNSCC patient. A represents the cell line was established from primary or first time excision, B represents the cell line was established from advanced or secondary excision. [file 12967_2017_1289_MOESM1_ESM.docx]

Table S1. Information of paired UMSCC cell lines

| UMSCC cell line | Description |
| --- | --- |
| 10A | from the larynx at the time of laryngectomy |
| 10B | from a submental lymph node metastasis |
| 11A | from epiglottis primary |
| 11B | supraglottic persistant |
| 14A | wide local excision after excisional biopsy |
| 14B | recurrence after surgery and radiation |
| 17A | from the endolarynx (supraglottis, primary) |
| 17B | from tumor extending outside the thyroid cartilage |
| 22A | from the primary site (hypopharynx primary) |
| 22B | from a lymph node metastasis(neck) |
| 74A | syrgical resection after chemotherapy and radiation |
| 74B | second surgery for persistent cancer |
| 83A | from buccal mucosa |
| 83B | from buccal mucosa |
