# Supplementary material for: Over-expression of BAG-1 in head and neck squamous cell carcinomas (HNSCC) is associated with cisplatin-resistance
Source: J Transl Med. 2017 Sep 6;15:189. doi: 10.1186/s12967-017-1289-2 (PMC5588726; doi:10.1186/s12967-017-1289-2)
Supplement: Supplementary file 2 — Additional file 2: Figure S1. Differential expression analysis of relapsed and local cell lines. T-tests were used to identify differentially expressed genes between relapse and primary cell lines. 739 genes (unadjusted p value <0.05) were shown. [file 12967_2017_1289_MOESM2_ESM.pdf]

Additional file 2: Figure S1

A

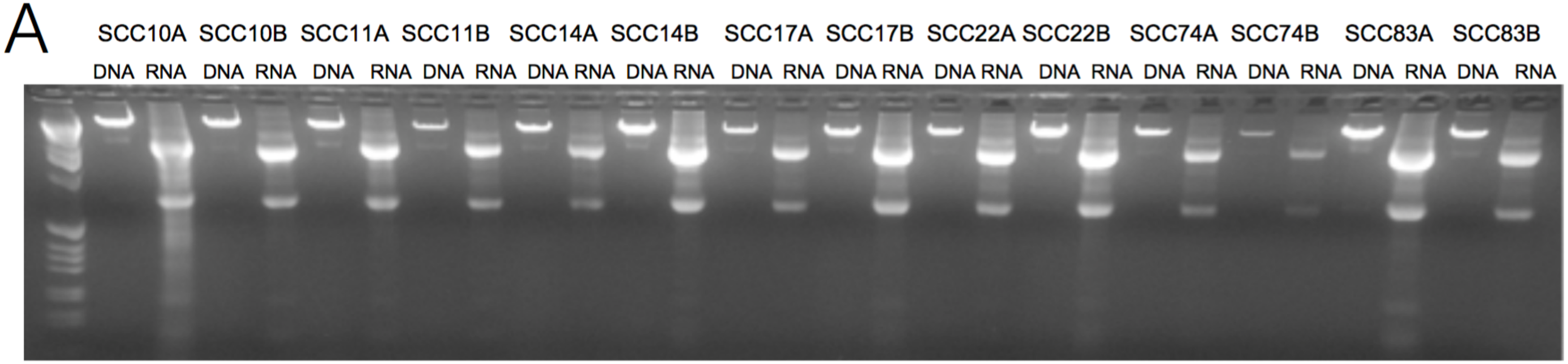

B

Top Diseases and Bio Functions

Diseases and Disorders

Name	p-value range	# Molecules
Cancer	2.47E-02 - 2.79E-05	146
Hematological Disease	2.47E-02 - 2.79E-05	91
Immunological Disease	2.47E-02 - 2.79E-05	89
Organismal Injury and Abnormalities	2.47E-02 - 2.79E-05	193
Inflammatory Disease	2.47E-02 - 2.87E-04	18

Molecular and Cellular Functions

Name	p-value range	# Molecules
Cell Death and Survival	2.47E-02 - 6.07E-04	6
Cell-To-Cell Signaling and Interaction	2.47E-02 - 6.07E-04	10
Cellular Compromise	2.47E-02 - 6.07E-04	9
Small Molecule Biochemistry	2.47E-02 - 6.07E-04	17
Cell Morphology	2.47E-02 - 3.06E-03	15
